# Hyponatremia in COVID-19 Is Not Always Syndrome of Inappropriate Secretion of Antidiuretic Hormone (SIADH): A Case Series

**DOI:** 10.7759/cureus.30939

**Published:** 2022-10-31

**Authors:** Muhammad M Javaid, Jocelyn Shan, Rachel Frederick, Reinhardt Dreyer, James J Gome

**Affiliations:** 1 Medicine, Monash University, Melbourne, AUS; 2 Medicine, Deakin University, Warrnambool, AUS; 3 Medicine, Royal Hobart Hospital, Hobart, AUS; 4 Medicine, St. Vincent's Hospital, Melbourne, AUS; 5 Medicine, South West Healthcare, Warrnambool, AUS

**Keywords:** thiazide diuretics, sodium, syndrome of inappropriate antidiuretic hormone secretion, siadh, hyponatraemia, covid-19

## Abstract

Hyponatremia is a common complication in COVID-19-positive patients and is associated with significant mortality and morbidity. Several cases of COVID-19-related hyponatremia secondary to the Syndrome of Inappropriate Secretion of Antidiuretic Hormone (SIADH) have been reported in the literature, which might suggest that SIADH is almost always the underlying cause of hyponatremia in COVID-19 infections. However, COVID-19-related hyponatremia can have diverse underlying etiologies, similar to hyponatremia in non-COVID-19 patients, and requires a thorough assessment to reach a correct diagnosis and implement appropriate management.

## Introduction

Since its initial identification in Wuhan, China, in December 2019, much has been learned about the epidemiology, presentation, and management of Coronavirus Disease 2019 (COVID-19). Although predominantly a respiratory tract infection, ranging from mild to severe disease caused by the novel Severe Acute Respiratory Syndrome Coronavirus 2 (SARS-CoV-2), the condition's effect on other organs has been well-described and can often result in multisystem illness [1].

Disorders of sodium balance are common in COVID-19, with both hypernatremia and hyponatremia reported in the literature [2]. Hyponatremia is one of the commonest electrolyte abnormalities affecting hospitalized patients and a significant cause of morbidity and mortality. Serum sodium (Na) levels of less than 135 mEq/L have been reported in 10%-45% of patients with COVID-19 in different studies with illnesses ranging from mild to severe [2,3].

In COVID-19 infections, hyponatremia can be a marker of the severity of the underlying pulmonary disease and an independent risk factor for intensive care admissions and mechanical ventilation [2,4]. Uncorrected COVID-19-associated hyponatremia after 72 to 96 hours of hospitalization has been associated with higher mortality [5].

While the Syndrome of Inappropriate Antidiuretic Hormone Secretion (SIADH) is thought to be the primary etiology in many cases [6-10], the underlying pathophysiology driving COVID-19-related hyponatremia can be varied, and a broader differential should be considered while assessing such patients. A thorough and systematic assessment is required to pinpoint the correct etiology and instigate the appropriate corrective therapy.

We share our experience of three COVID-19-positive patients with hyponatremia who presented within a short period to a regional hospital during the early stage of the third Omicron wave of COVID-19 infections in Victoria, Australia. All three patients had different underlying etiology for hyponatremia and thus required different treatment strategies.

## Case presentation

Case 1

An 81-year-old man presented with a six-day history of coryzal symptoms in keeping with COVID-19 illness. This was associated with increased confusion, lethargy, and fatigue. He was a known primary contact of his wife, who had contracted COVID-19. His past medical history was significant for immunosuppression from enzalutamide therapy for metastatic prostate carcinoma, Charcot Marie Tooth Disease, cervical stenosis requiring C3-6 laminectomy, a permanent pacemaker for complete heart block, and hypertension. He was a non-smoker and non-alcohol drinker. His regular medications included a calcium channel blocker, angiotensin receptor blocker, enzalutamide, and four-monthly leuprorelin acetate injections.

He tested positive for COVID-19 on rapid antigen self-testing (RAT), which was confirmed by a polymerase chain reaction (PCR) test. The patient sustained a mild COVID-19 illness, not requiring disease-modifying therapies throughout his admission. However, his condition was complicated by hyponatremia at presentation with a serum Na nadir of 124 mEq/L. He was clinically euvolemic on examination, with a blood pressure of 128/88 mmHg and a heart rate of 91 beats per minute. He was afebrile and had an oxygen saturation of 95% on room air. His hemoglobin, total white cell, neutrophil, and platelet counts were normal. A chest x-ray was not significant. Renal and liver function testings were within the normal range. Serum cortisol and thyroid function tests were normal. Serum osmolality was low at 258 mOsm/kg with an inappropriately elevated urinary osmolality of 351 mOsm/kg and urinary Na of 122 mEq/L, keeping with SIADH. He was fluid-restricted to 1 liter/day, and his serum Na gradually improved to 130 mEq/L before discharge.

Case 2

A 92-year-old man presented with a four-day history of persistent nausea, headache, dizziness, unsteadiness, and poor oral intake. He had close contact with a COVID-19-positive patient 10 days before admission. His only respiratory symptom was that of a mild cough. He had significant comorbidities, including hypertension, hyperlipidemia, ischemic heart disease with previous cardiac bypass, transient ischemic attacks, atrial fibrillation, asthma, gastroesophageal reflux, osteoporosis, ascending aortic aneurysm repair. He was a non-smoker and non-alcohol drinker with no recent travel history and no history of immunosuppression. His regular medications included a thiazide diuretic, novel anticoagulant, calcium channel blocker, angiotensin-converting enzyme inhibitor, statin, and vitamin D supplementation.

On examination, the patient was clinically hypovolemic. He was alert and orientated with a Glasgow Coma Scale (GCS) of 15. All other observations were within normal limits. He was found positive for COVID-19 infection with positive PCR. Initial blood tests showed that he had severe hyponatremia with a serum Na of 111 mEq/L on a background of normal serum Na of 136 mEq/L three months prior. Hemoglobin, total white cell, neutrophil, and platelet counts were normal. Renal and liver function testings were within the normal range. He was hypokalemic with serum potassium (K) of 2.9 mEq/L. Chest x-ray revealed no significant abnormality. Serum osmolality was low at 239 mOsm/kg with a urinary osmolality of 484 mOsm/kg and elevated spot urine Na at 102 mEq/L, affected by the thiazide diuretic.

His hyponatremia was primarily thought to be secondary to volume depletion due to a combination of thiazide diuretic and poor oral intake. The patient was admitted to the intensive care unit, where a single 100 mL bolus of hypertonic 3% saline was administered, correcting serum sodium to 114 mEq/L. Subsequently, he received fluid resuscitation with 0.9% saline until he was clinically euvolemic. The patient also received an intravenous potassium replacement. His thiazide diuretic was ceased indefinitely. Hyponatremia gradually improved, and he was eventually discharged symptom-free with a serum Na of 129 mEq/L.

Case 3

A 53-year-old previously healthy woman presented with acute onset confusion and an altered conscious state. Her husband described a one-day history of headaches and profuse diarrhea. He found her unresponsive the next day and immediately called ambulance services. She had a history of mild hypertension, for which she was not on any regular anti-hypertensives. She lived at home with her husband and worked full-time in an abattoir. She was a non-smoker and non-alcohol drinker with no recent travel history. The remainder of her systems review was unremarkable.

On arrival, she was drowsy with an initial GCS of 8. She had an oxygen saturation of 100% on room air. She was clinically hypovolemic and hypotensive with a blood pressure of 95/75 mmHg. COVID-19 RAT and PCR testings returned positive. Initial investigations revealed severe hyponatremia with serum Na of 111 mEq/L. Hemoglobin, total white cell, neutrophil, and platelet counts were normal. Renal function and liver function testings were within the normal range. Thyroid function testing and serum cortisol were within normal limits. The chest radiograph showed no abnormalities, and brain computed tomography (CT) and magnetic resonance imaging (MRI) demonstrated no abnormalities to explain the patient's symptoms. The cerebrospinal fluid examination was within normal limits. Fecal microscopy and culture were negative, as was *Clostridium difficile* toxin.

Due to the neurological manifestations of hyponatremia, hypertonic 3% saline was administered in the Emergency Department to raise the serum Na within a safe range. Her serum Na initially rose rapidly to 127 mEq/L, and intravenous therapy was changed to 5% dextrose. Following this, serum Na dropped to 113 mEq/L again, and the decision was made to change the fluid resuscitation to 0.9% normal saline. The patient's serum Na and conscious state gradually improved with appropriate intravenous volume replacement. She had a GCS of 15 and serum Na of 138 mEq/L at discharge time. Her diarrhea, which was thought to be a gastrointestinal manifestation of COVID-19 infection, and the likely cause of her symptomatic hyponatremia, also steadily improved during admission. Table 1 summarizes the key features of the three cases.

**Table 1:** Summary of clinical cases of hyponatremia AAA = abdominal aortic aneurysm, GORD = gastroesophageal reflux disease, GCS = Glasgow coma scale, Na = sodium, SIADH = syndrome of inappropriate secretion of antidiuretic hormone, TIA = transient ischemic attack

Case	Age/ Sex	Comorbidities	Serum Na	Clinical presentation	Associated drugs	Volume status	Cause	Treatment	Outcome
1	81/M	Prostate ca	124 mEq/L	Confusion	None	Euvolemic	SIADH	Fluid restriction	Full recovery
		Cervical stenosis		Lethargy					
		Permanent pacemaker		Fatigue					
		Hypertension							
2	92/M	Hypertension	111 mEq/L	Dizziness	Hydrochlorothiazide	Hypovolemic	Diuretic	Fluid and solute resuscitation	Full recovery
		Dyslipidemia		Nausea			Poor oral intake	Hydrochlorothiazide ceased	
		TIAs		Headache					
		Atrial fibrillation							
		Asthma							
		GORD							
		Osteoporosis							
		AAA repair							
3	53/M	Hypertension	111 mEq/L	Altered GCS	None	Hypovolemic	Diarrhea	Fluid and solute resuscitation	Full recovery
				Confusion			Poor oral intake		
				Headache					

## Discussion

Several cases of hyponatremia secondary to SIADH in COVID-19 patients have been reported in the literature [6-10], with most of these cases being attributed to pneumonia as the underlying cause of SIADH [7-10]. However, others have also reported the development of SIADH in the absence of pneumonia [6]. Production of inflammatory cytokines in COVID-19 infections, such as interleukin-6, resulting in direct stimulation of nonosmotic release of antidiuretic hormone (ADH) and cytokine-mediated lung injury inducing inappropriate ADH release via hypoxic pulmonary vasoconstriction pathway are considered possible underlying mechanisms in such cases [7,11]. From the reported cases, one might extrapolate that in COVID-19 patients, SIADH might be the sole cause of hyponatremia. However, our experience suggests that in the context of COVID-19 infection, the causes of hyponatremia remain diverse. Such cases should be carefully evaluated for an accurate diagnosis rather than an assumption of SIADH. Identifying the correct etiology of hyponatremia is essential for appropriate management, which varies according to the underlying cause.

Our first patient had clinical and biochemical features consistent with SIADH and improved with fluid restriction. On the other hand, our second patient was clinically hypovolaemic and was on long-term thiazide diuretics. Although the serum and urine osmolalities were consistent with SIADH, the patient did not fulfil the diagnostic criteria of SIADH (Table 2).

**Table 2:** Diagnostic criteria of SIADH Na = sodium, SIADH = syndrome of inappropriate secretion of antidiuretic hormone

Diagnostic criteria
Euvolemic hyponatremia
Low serum osmolality (< 280 mOsm/kg)
Inappropriately elevated urine osmolality (> 100 mOsm/kg)
High urine Na (> 30 mEq/L)
Absence of thyroid disorder
Absence of adrenal disorder
Absence of current diuretic use
Absence of any other cause of hyponatremia

Thiazide diuretics interfere with renal water excretion resulting in inappropriately high urine osmolality. As hyponatremia was considered secondary to thiazides, the patient was treated with solute replacement and withdrawal of the offending medication, which led to clinical improvement. Similarly, our third patient was volume-depleted due to diarrhea, the most likely cause of her hyponatremia. The patient responded well to adequate fluid and solute resuscitation.

Serum Na concentration is primarily a measure of plasma water content rather than the Na balance and is regulated by changes in water intake and excretion. Serum Na accounts for 85% of extracellular fluid osmolality and is typically maintained within a narrow range of 135-145 mEq/L with a corresponding serum osmolality of 280-295 mOsm/kg. Any change in serum osmolality is sensed by osmoreceptors in the hypothalamus, influencing the release of ADH and thirst. ADH regulates free water excretion through cortical and medullary collecting ducts in kidneys, resulting in concentrated or dilute urine.

Water retention leads to the suppression of ADH and the production of dilute urine (urine osmolality below 100 mOsm/kg), preventing hyponatremia development. On the other hand, plasma hyperosmolality stimulates thirst leading to increased water intake, which is the primary protective mechanism against water deficit and hypernatremia. ADH secretion is also increased appropriately in such situations resulting in concentrated urine. Serum Na concentration abnormalities occur when the osmoregulation is disrupted. Hyponatremia occurs when too much water cannot be excreted, and conversely, hypernatremia occurs when there is too little water that cannot be replaced [2,12].

True hyponatremia is almost always hypotonic (serum osmolality < 280 mOsm/kg). Isotonic (serum osmolality 280-295 mOsm/kg), or pseudohyponatremia, often results from severe hypertriglyceridemia, hyperproteinemia (multiple myeloma, monoclonal gammopathies, or intravenous immunoglobulin administration), or severe hypercholesterolemia (primarily associated with primary biliary cirrhosis). This is mainly due to an error in serum Na measurement when using an analyzer that measures Na per volume of plasma rather than per volume of water. Hypertonic hyponatremia (serum osmolality > 295 mOsm/kg) is often due to an osmotic active substance in the circulation such as glucose, mannitol, glycine, or radiocontrast agent resulting in the movement of water from intracellular to intravascular space [13]. Although not always necessary, measurement of serum osmolality and a detailed history can help to narrow down the differential diagnosis (Table 3).

**Table 3:** Causes of hyponatremia according to serum osmolality SIADH = syndrome of inappropriate secretion of antidiuretic hormone

Hypotonic	Isotonic	Hypertonic	
Hypovolemic (Gastrointestinal losses, dermal losses, renal losses, third spacing)	Hyperproteinemia (Multiple myeloma, monoclonal gammopathies, intravenous immunoglobulin administration)	Hyperglycemia	
		Other solutes (Mannitol, glycine, radiocontrast agents)	
Euvolemic (Drugs, SIADH, hypothyroidism, hypopituitarism, primary polydipsia)	Hypercholesterolemia (Primary biliary cirrhosis)		
	Hypertriglyceridemia		
Hypervolemic (Cardiac failure, liver cirrhosis, advanced kidney disease, IV therapy)			

True (hypotonic) hyponatremia can be subdivided into hypovolemic, euvolemic, or hypervolemic hyponatremia. Although at times difficult, careful assessment of a patient's volume status with detailed history and clinical examination may help reach the correct diagnosis (Table 4).

**Table 4:** Causes of hyponatremia according to volume status IV = intravenous, SIADH = syndrome of inappropriate secretion of antidiuretic hormone

Hypovolemic	Euvolemic	Hypervolemic	
Gastrointestinal losses (Diarrhea, vomiting)	Drugs (Carbamazepine, antipsychotics, antidepressants)	Cardiac failure	
		Liver cirrhosis	
Renal losses (Diuretics, adrenal insufficiency, renal tubular acidosis)			
		Advanced kidney disease	
		Iatrogenic (e.g., IV therapy)	
	SIADH		
	Hypothyroidism		
	Hypopituitarism		
	Primary polydipsia		
Dermal losses (Perfuse sweating, burns)			
Third spacing (Pancreatitis, bowel obstruction)			

Hypovolemic hyponatremia can occur due to gastrointestinal (diarrhea or vomiting), renal (diuretics, cerebral salt wasting, or adrenal insufficiency), or dermal (perfuse sweating or burns) salt and water loss. In these cases, patients have both water and total body sodium deficit. However, the sodium deficit exceeds the water deficit resulting in relative free water excess and hypotonic hyponatremia. Hypervolemic hyponatremia is commonly seen in patients with cardiac failure, liver cirrhosis, nephrotic syndrome, and advanced chronic kidney disease. Patients with hyponatremia secondary to SIADH, hypothyroidism, hypopituitarism, and primary polydipsia are usually euvolemic [2,12].

SIADH is the commonest cause of hyponatremia in hospitalized patients, accounting for 40%-50% of cases. The syndrome is diagnosed by the presence of euvolemic hyponatremia, low serum osmolality (<280 mOsm/kg), inappropriately elevated urine osmolality (>100 mOsm/kg), high urine Na (>30 mEq/L), and the absence of thyroid disorder, adrenal disorder, and current diuretic use [6]. SIADH is usually secondary to other abnormalities, including central nervous system disorders, pulmonary diseases, malignancies, infections, and medications [14]. It is important to remember that SIADH is primarily a diagnosis of exclusion, and other causes of hyponatremia must be excluded before making a diagnosis [12].

**Figure 1:**
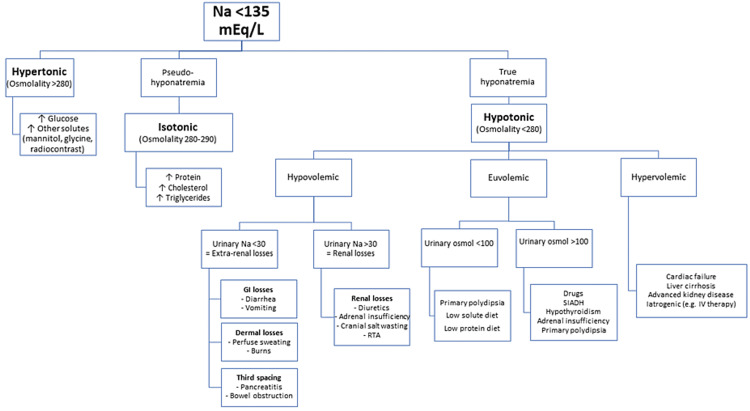
Flowchart for diagnosing hyponatremia IV = intravenous, Na = sodium, RTA = renal tubular acidosis, SIADH = syndrome of inappropriate secretion of antidiuretic hormone

Thiazide diuretics are an important cause of hyponatremia in hospitalized patients, which can sometimes be severe. Hyponatremia is often seen during the early stage of treatment with thiazides but can also occur after months or even years of therapy. Although hypovolemia-induced ADH secretion might be responsible for hyponatremia in some cases, most patients with thiazide-associated hyponatremia are euvolemic [15]. The contributing factors include increased water intake, cation depletion, impaired free water excretion, and sodium osmotic inactivation. The treatment involves a combination of the cessation of thiazide diuretics, cation replacement, and restriction of free water intake [15]. Figure 1 summarizes the steps that can help evaluate hyponatremia in most cases, including in patients with COVID-19 infections.

## Conclusions

Hyponatremia is a common complication of COVID-19 infections and is associated with increased morbidity and mortality. Our experience shows that the underlying etiology of hyponatremia in patients with COVID-19 infection can be varied, including SIADH, gastrointestinal losses, and concurrent use of diuretic treatment. Hyponatremia in COVID-19 patients should be evaluated in the same way as in non-COVID-19 patients to pinpoint an accurate diagnosis which is crucial for proper management.
